# A 4-study replication of the moderating effects of greed on socioeconomic status and unethical behaviour

**DOI:** 10.1038/sdata.2016.120

**Published:** 2017-01-31

**Authors:** Anjana Balakrishnan, Paolo A. Palma, Joshua Patenaude, Lorne Campbell

**Affiliations:** 1 Western University, Department of Psychology, London, N6A 3K7 Ontario, Canada

**Keywords:** Human behaviour, Ethics

## Abstract

Four replications of Piff and colleagues’ study examined the moderating effects of greed attitudes on the relationship between socio-economic status (SES) and unethical behaviour (Study 7). In the original study, the researchers found that both greed and SES predicted increased propensity to engage in unethical behavior. Furthermore, this association was moderated such that the effects of SES on unethical behaviour were no longer present in the greed prime condition versus the neutral condition. In replication 1 of the original study main effects of greed attitudes and SES were found, but no interaction was found. Main effects for greed emerged in replications 3 and 4. However no main effects for SES or interactions emerged for replications 2–4. A meta-analysis was conducted with all replications and the original study, and found no moderating effect of greed on the relationship between SES and unethical behavior.

## Background & Summary

How do the rich differ from the rest of society? This question has been at the forefront of public interest, leading to various media outlets (e.g., Business Insider, Time, Wired) and books (e.g., How Rich People Think by Steve Siebold) focusing on this very topic. One theme converged on by the popular media is that compared to those in the middle and lower class the highly affluent consider selfishness as a virtue^1,2^. This lay belief was tested in a series of studies conducted by Piff, Stancato, Côté, Mendoza-Denton, and Keltner^3^. Through various naturalistic and experimental methods, Piff *et al.*^3^ found that high socioeconomic status (SES) individuals were more likely to engage in unethical self-serving behaviors compared to low SES individuals. Specifically, high SES individuals were found to be more likely to break the law while driving (Study 1 and 2), exhibit unethical decision-making tendencies (Study 3), steal valued goods (Study 4), lie in negotiations (Study 5), cheat to increase their chances at winning (Study 6) and endorse unethical behavior (Study 7). Importantly however, in their final study (Study 7), Piff *et al.*^3^ found that the difference in unethical behavior between high and low SES individuals was moderated by attitudes toward greed. That is, when individuals were prompted to think about the benefits of acting greedily, individuals both high and low in SES indicated a similar propensity to engage in unethical behavior.

Piff *et al.*^3^ study on SES, greed, and unethical behavior, has had a significant impact both in and out of academia. As of mid-2016, their paper has been cited over 300 times and has been covered by numerous popular media outlets such as Time^4^ and Wired^5^. Furthermore, their findings are not only consistent with lay theories espoused by popular media that rich people think differently than the rest of society^1,2^, but also suggest that thinking like a rich person (i.e., seeing greed as beneficial) will result in adopting behaviour similar to rich people. Despite this, there is evidence that the relation between SES and unethical behavior is not clear-cut as there is contrary evidence suggesting that high SES predicts prosocial behavior^5^.

The goal of the current project was to directly replicate Study 7 in Piff *et al.*^3^ and share the four replication datasets^3^. This study was chosen because it encapsulates the main idea regarding the association between greed and unethical behaviour but also tests the importance of attitudes toward greed in driving this association. Specifically, thinking like a rich person (i.e., greed is good) leads to acting like a rich person (i.e., no SES difference in propensity to engage in unethical behavior). Furthermore, a direct replication is warranted due to the paper’s high impact in academia as evidenced by the number of citations. The paper being cited at least 300 times since publication, and furthermore there is some evidence (both anecdotal examples and empirical research) suggesting that the relations between SES, greed, and unethical behavior is not definitive.

Four replication studies (three direct and one conceptual) were conducted in this multi-sample study^6^. In terms of study design, participants were provided with the same questionnaires and procedures employed by Piff *et al.*^3^ in an initial replication attempt. The second and third replications were identical to the first save for using additional exclusion criteria (i.e., excluding individuals with inappropriate and missing responses to priming questions). While replications 1–3 used participants from Amazon’s Mechanical Turk (MTurk), the replication 4 examined an undergraduate sample. Additionally, replication 4 was identical to replications 2 and 3 save for the inclusion of a question to assess parents’ jobs and income as an additional quality check to assess their self-reported SES. For each replication sample, regression analyses testing main effects of SES and a manipulation of attitudes toward greed, as well the interaction between these two variables, were conducted. Additionally, a meta-analysis of the unstandardized interaction coefficient (the primary test of the hypothesis) was conducted including data from the original study and each replication. All datasets, syntax, and usage files are publicly available for any future research on the OSF project page (Data Citation 1). Specific information about questionnaires and procedure as well as where to access data is provided in the Method section.

## Methods

### Pre-registration and replication report

The replications were conducted using the recommendations from Brandt *et al.*^7^ replication recipe. As a starting point, the corresponding author of the original study was contacted prior to data collection to ensure our methodology followed the original procedure as closely as possible. Second, a sample size at least 2.5 times the original was chosen in order to achieve high statistical power based on recommendations by Simonsohn^8^. Although we attempted to collect at least 2.5 times the original sample across all replications, we were unable to collect this sample for the undergraduate population due to resource constraints. Lastly, the project and a replication report outlining in detail the methodology and statistical approach for the current study were pre-registered and publicly disclosed on the Open Science Framework^9^. Although the authors aimed to directly replicate the original study, the present studies differ from the original in two ways. First, participants were compensated $0.30 in the original study. In order to remain competitive in the current offerings of MTurk, we compensated participants in the present study $0.50. Second, auxiliary analyses were pre-registered to examine the relations between SES, greed, and unethical behavior without controlling for covariates. Additionally, the final replication sought to examine the effects of greed and SES in a broader context by recruiting Canadian undergraduates from the University of Western Ontario to participate in an online study.

### Participants and recruitment

Participants for replications 1–3 were recruited from MTurk with an advertisement requesting them to participate in a ‘short task which involves assessing your attitudes on various issues’. Recruitment was limited to English-speaking participants who had an MTurk approval rating of 95%. In replication 3, there was a heavy skew of participants in the neutral-prime condition (*n*=166 versus *n*=93) which was due to a large amount of missing data. This missing data resulted from participants starting, but not completing any questions. It is unclear about why participants dropped out of the study, as participants did not answer any questions before dropping out, and as such this suggests that the skew may be random. This differential dropout pattern was only seen in replication 3. To address this skew in participant conditions, additional participants were recruited specifically for the greed-prime condition (*n*=47 after validation). For replication 4, participants were recruited for an online study from Western University’s undergraduate participant pool. This pool included students from an introductory psychology class and an introductory research methods course. After technical validation, a total of 983 participants were retained across the four studies (see Table 1 for exact details of the participants in each replication).

### Materials and procedure

Participants were randomly assigned to either the greed-is-good or the neutral-prime conditions. In the greed-is-good condition, participants were asked to ‘Please take a few minutes to think of ways in which acting greedily and pursuing your self-interest can be good. For example, being greedy, or prioritizing self-interest, may allow you to be successful and achieve your professional goals. Please think of three additional ways in which greed can be good and write them in the boxes below.’ In contrast, participants in the neutral-prime condition were asked to ‘Please take a few minutes to think the things you do in an average day. For example, one might go to work or spend time at the gym. Please think of three things that you do in an average day and write them in the boxes below.’ Three text boxes were presented below each of the prompts for the participants to write their answers. Once the participants completed their responses, they were able to continue towards the manipulation check.

The effect of the manipulation was assessed with a five-item scale assessing positive beliefs about greed^10^, and participants rated their agreement from 1 (strongly disagree) to 7 (strongly agree). Sample items include ‘Overall, greed is good’ and ‘It is good to be greedy.’ Items were presented all at once in random order. The manipulation check was immediately^11^ followed by a 12-item questionnaire assessing the participant’s propensity to engage in unethical behavior^3,12^ from 1 (very unlikely) to 7 (very likely). Questions ranged in severity, from sample items asking participants the likelihood they would ‘Use office supplies, Xerox machine, and stamps for personal use’ to ‘Make more money by deliberately not letting clients know about their benefits’. Each item was presented sequentially in the order reported in Piff *et al.*^3^ original study. Descriptive statistics for all the variables and reliability for the greed attitudes and unethical behavior scales are summarized in Tables 2 and 3 respectively.

After completing the main portion of the study, participants were asked to complete a demographics questionnaire which included questions about their sex (male, female), race/ethnicity (Select all that apply: European American, African American, Latino, East Asian/ Asian American, Native American, Other please specify), socio-economic status, political orientation, and religiosity, in that order. Religiosity was assessed with a single item asking participants ‘How religious are you’ from 1 (not at all religious) to 7 (deeply religious). Political orientation was assessed with a single item asking participants ‘What is your political orientation’ with responses ranging from 1 (extremely liberal) to 7 (extremely conservative). Lastly, the critical demographic variable, social class, was assessed using the MacArthur scale of subjective SES^3,12^ in which participants were asked to imagine a ladder with 10 rungs representing people’s standing in their local community, with higher rungs representing those who are better off than people in the lower rungs. Participants were asked to indicate which rung they would belong to in their society ranging from 1 (bottom rung) to 10 (top rung).

In all replications, participants were given an attention probe which asked what they wrote about in the beginning of the study (e.g., Greed is good, greed is bad, my day, the environment, politics). In the second replication onwards, participants were also asked if they participated in a similar survey and what they thought the purpose of the study was. In addition, the third replication asked participants about their job/career and annual income and the fourth asked participants about their parent’s job/ parent career and income. This extra information was used as additional data-quality checks to examine if participant responses on the MacArthur scale were reasonable based on either participant or participant jobs and income. No participants were removed based on these additional criteria. Complete questionnaires can be found on the OSF project page^9^.

### Code availability

The code is available for access on the OSF project page^9^. There are no restrictions to the use of this code. For clarification, a separate file explaining the meaning of each of the terms in the code files has been added to the OSF page. The data was analysed on SPSS version 23 and the meta-analysis was conducted on R using the *metafor* package^13^.

## Data Records

All data from replications 1 to 4 can be found on the OSF project page (see Tables 3,4 for details). The OSF project page contains SPSS data files, SPSS output files, SPSS syntax files, R code file for meta-analysis, and a file clarifying what the terms in the syntax and data files mean (i.e., meta-data).

## Technical Validation

The following steps were taken in order to ensure the quality of the data-sets used. Firstly, upon completion of data collection, the data files were examined, and participants with missing data and those who failed the manipulation check were removed. This procedure is consistent with that used in the original study by Piff *et al.*^3^. Based on the quality of written responses in replication 1 to the question that served as the manipulation, third-party coders blind to the condition qualitatively assessed the content of the participant’s responses (i.e., whether they were responding to their prompt) for replications 2–4. Cases in which coders were unable to agree with participant responses or when participant responses were discordant with the prompt were removed from analysis. Lastly, participants who were able to articulate the purpose of the study (i.e., examining how talking about ways in which greed is good facilitates propensity to engage in unethical behavior) were removed prior to analyses. A regression analysis was conducted with unethical behavior as the outcome variable, and with greed (coded 0=neutral, 1=greed-is-good) and SES (1=lowest SES xx, 10=highest SES) as predictors. Age, gender, religiosity, political orientation, and race were statistically controlled. Analyses were conducted with a listwise deletion on SPSS, resulting in no extra participants being excluded.

## Results

In each replication attempt, the main effects and interaction of greed prime and SES on unethical behavior were modeled using linear regressions, and age, ethnicity, sex, religiosity, and political orientation were statistically controlled as covariates across the four replications. Although we pre-registered analyses for the regression model without covariates, the pattern of results for the interaction did not differ regardless of inclusion of covariates for all replications, and thus was not reported in the final paper. Analysis of the regression models without covariates can be found in our output files on our data records. A manipulation check revealed that participants in the greed-is-good condition had more favourable attitudes towards greed compared to those in the neutral condition across all four replications at *P*<0.05.

### Mturk sample (Replications 1–3)

In replication 1, participants in the greed-is-good condition reported a higher propensity to engaging in unethical behaviour compared to controls, *b*=0.404, s.e.=0.125, *t*(263)=3.241, *P*=0.001. There was also a main effect of SES such that participants who reported higher SES reported lower levels of unethical behaviour, *b*=−0.126, s.e.=0.034, *t*(263)=−3.742, *P*<0.001. In contrast to Piff *et al.*^3^ original study, no interaction was found between SES and the greed prime, *b*=0.111, s.e.=0.066, *t*(263)=1.686, *P*=0.093. When we investigated the auxiliary analysis with no covariates, both SES, *b*=−0.167, s.e.=0.034, *t*(262)=−4.862, *P*<0.001; and greed prime, *b*=0.453, s.e.=0.132, *t*(262)=3.441, *P*=0.001, remained significant predictors of unethical behavior such that lower SES and greed prime predicted a greater propensity towards unethical behavior. The interaction in the auxiliary analysis remained non-significant, *b*=0.058, s.e.=0.069, *t*(262)=0.846, *P*=0.398.

In replication 2, neither SES, *b*=−0.011, s.e.=0.024, *t*(255)=−0.436, *P*=0.663, nor the greed priming condition, *b*=0.158, s.e.=0.095, *t*(255)=1.663, *P*=0.098, predicted propensity to engage in unethical behaviour. No interaction was found between SES and the greed prime, *b*=−0.060, s.e.=0.050, *t*(255)=−1.192, *P*=0.234. When we investigated the auxiliary analysis with no covariates, only the greed prime, b=0.198, s.e.=0.097, *t*(255)=2.034, *P*=0.043, was a significant predictor for unethical behavior such that lower greed prime predicted a greater propensity towards unethical behavior. SES was not a significant predictor, *b*=−0.007, s.e.=0.025*, t*(255)=−0.301, *P*=0.763. The interaction was not significant *b*=−0.087, s.e.=0.051, *t*(255)=−1.706*, P*=0.089.

In replication 3, SES was not significant, *b*=−0.023, s.e.=0.028, *t*(305)=−0.804, *P*=0.422, but the greed-priming condition predicted propensity to engage in unethical behaviour, *b*=0.191, s.e.=0.095, *t*(305)=2.021, *P*=0.044. No interaction was found between SES and the greed prime, *b*=0.008, s.e.=0.056, *t*(305)=0.143, *P*=0.887. When we investigated the auxiliary analysis with no covariates, only the greed prime, b=0.239, s.e.=0.096, *t*(262)=2.484, *P*=0.014, was a significant predictor for unethical behavior such that lower greed prime predicted a greater propensity towards unethical behavior. SES was not a significant predictor, *b*=−0.015, s.e.=0.029*, t*(262)=−0.519, *P*=0.604. The interaction was not significant *b*=0.016, s.e.=0.058, *t*(262)=0.268*, P*=0.789.

### Undergraduate sample (Replication 4)

The final replication found no main effects of SES, *b*=−0.018, s.e.=0.049, *t*(112)=−0.366, *P*=0.715, however a main effect for priming condition was found such that participants in the greed condition reported a higher propensity to engage in unethical behaviour, *b*=0.355, s.e.=0.159, *t*(112)=2.238, *P*=0.027. No interaction was found between SES and the greed prime, *b*=0.095, s.e.=0.100, *t*(112)=0.948, *P*=0.345. When we investigated the auxiliary analysis with no covariates, only the greed prime, *b*=0.364, s.e.=0.154, *t*(112)=2.364, *P*=0.020, was a significant predictor for unethical behavior such that lower greed prime predicted a greater propensity towards unethical behavior. SES was not a significant predictor, *b*=−0.018, s.e.=0.048, *t*(112)=−0.377, *P*=0.707. The interaction was not significant *b*=0.053, s.e.=0.097, *t*(112)=0.543, *P*=0.588.

### Meta-analysis

**Figure 1:**
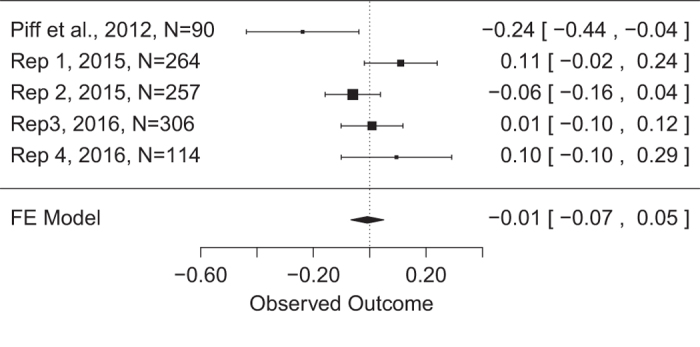
Meta-analysis of the unstandardized regression coefficients of the interaction between SES and greed prime across studies.

In order to generate the most precise estimate of the interaction effect of SES and attitudes toward greed on unethical behaviour, we conducted a fixed effects meta-analysis on the unstandardized regression coefficients obtained in the original and replication studies. Piff *et al.*^3^ reported a regression coefficient of *b*=−0.24, s.e.=0.18, *t*(84)=−2.34, *P*<0.03, but this combination of b and s.e. would not actually result in the reported t value (i.e., −0.24/0.18≠−2.34). We therefore contacted the corresponding author and obtained the following correct values: *b*=−0.238, s.e.=0.102, *t*(84)=−2.336, *P*=0.022 (note that the originally reported s.e. was incorrect). The metafor package^13^ for R was used to conduct the meta-analysis and generate the 95% CI for both the original and replication studies (Fig. 1). The weighted mean unstandardized coefficient was *b*=−0.01, 95% CI [−0.07, 0.05]. Based on the meta-analytic findings, the effect sizes of the interaction between priming condition and SES do not differ significantly from zero.

## Usage Notes

This data is all open access and free to use for any individuals who would like to use it for research purposes. This data may be of interest for those who wish to further investigate the associations between socioeconomic status, greed, and unethical behaviour. Across four independent replications investigating the interaction between greed and unethical behaviour, a near-zero effect size was found. In the current study, the findings did not suggest any significant differences from the null.

We originally pre-registered an analytic plan that included one primary regression model including both main effects and the interaction term. We deviated from this plan a little in order to report the main effects obtained from models that did not include the interaction term (reported in the above text). Tables 5,6 summarizes the original pre-registered main effects.

## Additional information

**How to cite this article:** Balakrishnan, A. *et al.* A 4-study replication of the moderating effects of greed on socioeconomic status and unethical behaviour. *Sci. Data* 4:160120 doi: 10.1038/sdata.2016.120 (2017).

**Publisher’s note:** Springer Nature remains neutral with regard to jurisdictional claims in published maps and institutional affiliations.

## Supplementary Material



## Figures and Tables

**Table 1:** Demographics.

**Replication**	***N***	**Priming Condition**		**Gender**		**Age**	
		**Greed**	**Neutral**	**Male**	**Female**	**M**	**s.d.**
1	264	113	151	113	151	35.56	12.33
2	257	95	162	106	151	34.92	11.56
3	306	141	165	145	161	37.17	12.41
4	114	48	66	49	65	18.97	1.15
Total	941	397	544	413	528		

**Table 2:** Descriptive Statistics for participants in the Neutral (A) and Greed (B) prime conditions.

**Replication**	**Greed Attitudes**		**SES**		**Unethical behavior**		**Political Orientation**		**Religiousness**	
	**M**	**s.d.**	**M**	**s.d.**	**M**	**s.d.**	**M**	**s.d.**	**M**	**s.d.**
	A									
1	2.825	1.360	5.106	1.866	1.910	0.979	3.250	1.702	3.350	2.195
2	2.707	1.208	4.722	1.912	1.789	0.725	3.51	1.673	3.19	2.102
3	2.570	1.088	4.982	1.567	1.749	0.785	3.321	1.739	3.358	2.258
4	2.982	0.958	6.606	1.616	1.902	0.800	3.180	1.391	2.890	1.931
	B									
1	3.680	1.328	5.327	1.957	2.400	1.249	3.480	1.643	3.350	2.100
2	3.383	1.272	4.779	1.897	1.988	0.798	3.33	1.765	3.13	2.194
3	3.553	1.198	5.141	1.750	1.990	0.890	3.418	1.695	3.170	2.308
4	3.629	0.968	6.813	1.593	2.269	0.815	3.310	1.461	3.040	1.935

**Table 3:** Cronbach’s Alphas.

**Replication**	**Greed attitudes (*****n*****=5 items)**	**Unethical behavior (*****n*****=12 items)**
1	0.898	0.919
2	0.885	0.823
3	0.878	0.871
4	0.840	0.856

**Table 4:** Metadata Records.

**Source**	**Subjects**	**Sample size**	**Protocol**	**Experimental manipulation**	**Data**
Replication Data: Replication 1	MTurk Participants	*N*=264	Questionnaire	Presence/Absence of Greed Prime	https://osf.io/jqrma/
Replication Data: Replication 2	MTurk Participants	*N*=257	Questionnaire	Presence/Absence of Greed Prime	https://osf.io/jqrma/
Replication Data: Replication 3	MTurk Participants	*N*=306	Questionnaire	Presence/Absence of Greed Prime	https://osf.io/jqrma/
Replication Data: Replication 4	Undergrad Participants	*N*=114	Questionnaire	Presence/Absence of Greed Prime	https://osf.io/jqrma/

**Table 5:** Participants Removed.

**Replication number**	Removed due to quality check failure*	**Removed due to failed attention probe**
1	2	28
2	28	14
3	34	17
4	19	34

*Quality check refers to i) participants who did not provide an answer to a variable which precluded their data from analysis ii) participants whose manipulation condition could not be accurately discerned by coders from their responses, suggesting that participants did not pay attention to the condition's prompt or iii) participants who guessed our hypothesis.

**Table 6:** Main effects from the full regression model including the interaction term (i.e., conditional main effects).

	**Greed Prime**					**Socioeconomic Status**				
	**b**	**s.e.**	**t**	**df**	**P-*value***	**b**	**s.e.**	**t**	**df**	**P-*value***
Rep 1	−0.178	0.367	−0.485	262	0.628	0.075	0.045	1.668	262	0.097
Rep 2	0.447	0.260	1.718	255	0.087	0.033	0.030	1.073	255	0.284
Rep 3	0.151	0.298	0.507	304	0.613	0.019	0.040	0.462	304	0.644
Rep 4	−0.282	0.690	−0.408	112	0.684	−0.021	0.065	−0.331	112	0.741
