# Corrigendum: A 4-study replication of the moderating effects of greed on socioeconomic status and unethical behaviour

**DOI:** 10.1038/sdata.2017.119

**Published:** 2017-09-05

**Authors:** Anjana Balakrishnan, Paolo A. Palma, Joshua Patenaude, Lorne Campbell

**Keywords:** Human behaviour, Ethics

*Scientific Data* 4:160120 doi: 10.1038/sdata.2016.120 (2017); Published 31 January 2017; Updated 5 September 2017.

It was brought to the authors' attention that there was an error in the SPSS syntax for the regression models analyzing the main effects. We had recoded the SES variable so that higher numbers corresponded to a higher SES. We did not use the recoded value in our syntax for the main effects model, resulting in the regression coefficient having the opposite sign. In addition, the description of the directionality for the greed prime on unethical behaviour was incorrectly reported in the results section. Overall the corrections do not affect the main conclusion drawn from the study: Across four independent replications investigating the interaction between greed prime and SES on unethical behaviour, a near-zero effect size was found. We regret the error in reporting the main effects. Though this error did not affect the focal hypothesis, we believe it is important to address this issue for the sake of open science, and in case these findings are used in future studies or meta-analyses. We are grateful to Dr Piff and his colleagues for bringing this to our attention.

The OSF has been updated with the correct syntax and outputs for all four replications (https://osf.io/92qkx/). The original documents have been retained and put in a separate archive folder on the OSF page to minimize the confusion.

**Correction: Paragraph 1 of section “Mturk sample (Replication 1–3)”**

- In the second sentence, a negative association between SES and unethical behaviour was incorrectly reported (*b*=−0.126, s.e.=0.034, *t*(263)=−3.742). The correct values are *b*=0.126, s.e.=0.034, *t*(263)=3.742. That is, participants who reported higher SES reported a higher propensity to engage in unethical behaviour.
- In the fourth sentence, a negative association between SES and unethical behaviour (*b*=−0.167, s.e.=0.034, *t*(262)=−4.862was incorrectly reported. The correct values for SES are *b*=0.167, s.e.=0.034, *t*(262)=4.862. That is, participants who reported a higher SES reported a higher propensity to engage in unethical behavior.

**Correction: Paragraph 2 of section “Mturk sample (Replication 1–3)”**

- In the first sentence, the relationship between SES and unethical behaviour was incorrectly reported with a negative sign (*b*=−0.011, s.e.=0.024, *t*(255)=−0.436). The correct values are *b*=0.011, s.e.=0.024, *t*(255)=0.436. The relationship remained non-significant.
- In the third sentence where it reads “…such that lower greed prime…”, the sentence should read “…such that greed prime…”
- In the penultimate sentence, the relationship between SES and unethical behaviour was incorrectly reported with a negative sign *b*=−0.007, s.e.=0.025*, t*(255)=−0.301. The correct values are *b*=0.007, s.e.=0.025*, t*(255)=0.301. The relationship remained non-significant.

**Correction: Paragraph 3 of section “Mturk sample (Replication 1–3)”**

- In the first sentence, the relationship between SES and unethical behaviour was incorrectly reported with a negative sign (*b*=−0.023, s.e.=0.028, *t*(305)=−0.804). The correct values are *b*=0.023, s.e.=0.028, *t*(305)=0.804. The relationship remained non-significant.
- In the third sentence where it reads “…such that lower greed prime…”, the sentence should read “…such that greed prime…”
- In the penultimate sentence, the relationship between SES and unethical behaviour was incorrectly reported with a negative sign *b*=−0.015, s.e.=0.029*, t*(262)=−0.519. The correct values are *b*=0.015, s.e.=0.029*, t*(262)=0.519. The relationship remained non-significant.

**Correction for section “Undergraduate Sample (Replication 4)”**

- In the first sentence, the relationship between SES and unethical behaviour was incorrectly reported with a negative sign (*b*=−0.018, s.e.=0.049, *t*(112)=−0.366). The correct values are *b*=0.018, s.e.=0.049, *t*(112)=0.366. The relationship remained non-significant.
- In the third sentence where it reads “…such that lower greed prime…”, the sentence should read “…such that greed prime…”

In the penultimate sentence, the relationship between SES and unethical behaviour was incorrectly reported with a negative sign (*b*=−0.018, s.e.=0.048, *t*(112)=−0.377). The correct values are *b*=0.018, s.e.=0.048, *t*(112)=0.377. The relationship remained non-significant.

